# Supplementary figures and images for: SGO2 does not play an essential role in separase inhibition during meiosis I in mouse oocytes
Source: PLoS Biol. 2025 Apr 23;23(4):e3003131. doi: 10.1371/journal.pbio.3003131 (PMC12017502; doi:10.1371/journal.pbio.3003131)

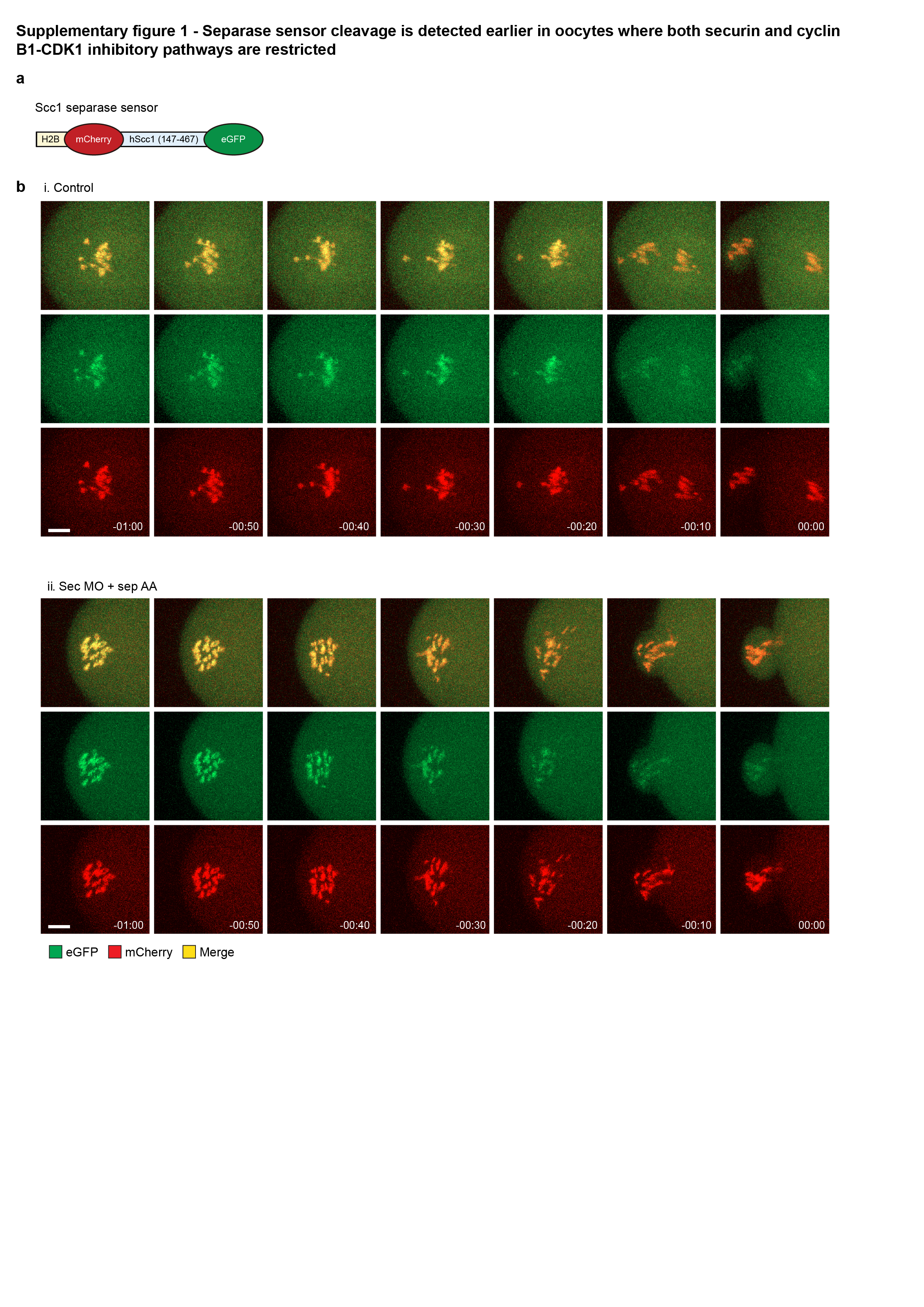

Supplement: S1 Fig — (a) Schematic diagram showing the H2B-mCherry-hScc1-eGFP separase sensor. (b) Representaive images of (i) control and (ii) securin MO + separase AA oocytes at 10-min intervals over 1 h prior to PB1 extrusion. Panels show the mCherry signal which remains histone-bound, the eGFP signal which dissociates into the cytoplasm on sensor cleavage, and their overlay. Notably in (ii), securin MO + separase AA oocytes, the eGFP signal begins to decrease approximately 30 min earlier than in (i), control oocytes. In this example, the majority of chromosomes are extruded in the polar body. (TIF) [file pbio.3003131.s001.tif]

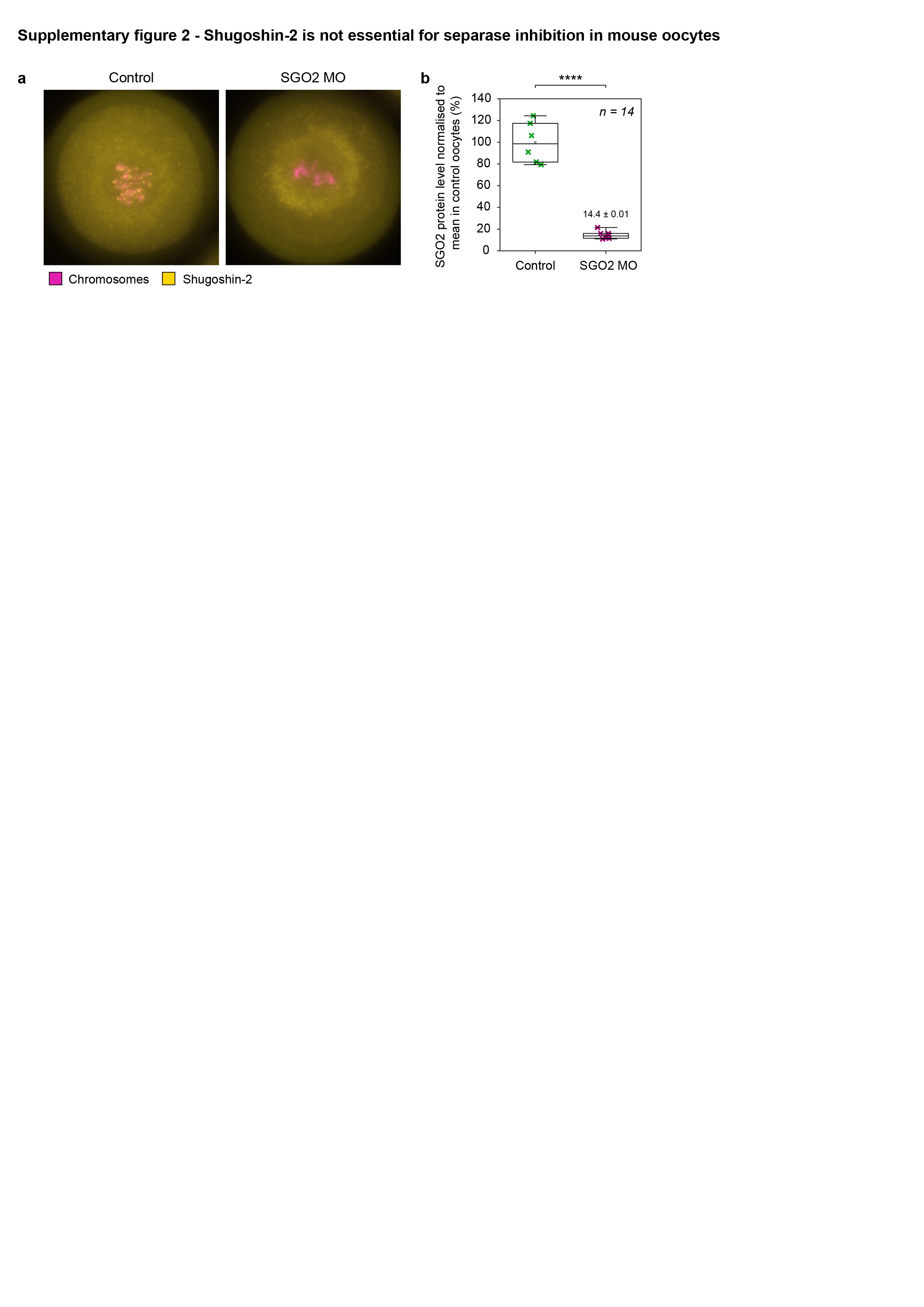

Supplement: S2 Fig — (a) Representative images of control and SGO2 MO oocytes collected at 3 h post-NEBD and immunostained for SGO2. (b) Quantification of SGO2 protein levels in control (green crosses, n = 6) and SGO2 MO (magenta crosses, n = 8) oocytes normalized to the mean SGO2 protein level in control oocytes. In panel (b): ****P < 0.0001, ***P < 0. 001, **P < 0.01, *P < 0.1, n.s. = non-significant. Significance was calculated by unpaired t test. All source data can be found in the S1 Data spreadsheet. (TIF) [file pbio.3003131.s002.tif]
